# Parallel Nanoimprint Forming of One-Dimensional Chiral Semiconductor for Strain-Engineered Optical Properties

**DOI:** 10.1007/s40820-020-00493-3

**Published:** 2020-08-08

**Authors:** Yixiu Wang, Shengyu Jin, Qingxiao Wang, Min Wu, Shukai Yao, Peilin Liao, Moon J. Kim, Gary J. Cheng, Wenzhuo Wu

**Affiliations:** 1 School of Industrial Engineering, Purdue University, West Lafayette, IN 47907 USA; 2 Flex Laboratory, Purdue University, West Lafayette, IN 47907 USA; 3 Department of Materials Science and Engineering, University of Texas at Dallas, Richardson, TX 75080 USA; 4 School of Materials Engineering, Purdue University, West Lafayette, IN 47907 USA; 5 Birck Nanotechnology Center, Purdue University, West Lafayette, IN 47907 USA; 6 Regenstrief Center for Healthcare Engineering, Purdue University, West Lafayette, IN 47907 USA

**Keywords:** Chiral semiconductor, Nanowires, Nanoimprinting, Strain engineering, Optical property

## Abstract

The low-dimensional, highly anisotropic geometries, and superior mechanical properties of one-dimensional (1D) nanomaterials allow the exquisite strain engineering with a broad tunability inaccessible to bulk or thin-film materials. Such capability enables unprecedented possibilities for probing intriguing physics and materials science in the 1D limit. Among the techniques for introducing controlled strains in 1D materials, nanoimprinting with embossed substrates attracts increased attention due to its capability to parallelly form nanomaterials into wrinkled structures with controlled periodicities, amplitudes, orientations at large scale with nanoscale resolutions. Here, we systematically investigated the strain-engineered anisotropic optical properties in Te nanowires through introducing a controlled strain field using a resist-free thermally assisted nanoimprinting process. The magnitude of induced strains can be tuned by adjusting the imprinting pressure, the nanowire diameter, and the patterns on the substrates. The observed Raman spectra from the chiral-chain lattice of 1D Te reveal the strong lattice vibration response under the strain. Our results suggest the potential of 1D Te as a promising candidate for flexible electronics, deformable optoelectronics, and wearable sensors. The experimental platform can also enable the exquisite mechanical control in other nanomaterials using substrate-induced, on-demand, and controlled strains.

## Highlights

- Exquisite strain engineering in 1D chiral semiconductor.
- Facile nanoimprinting induced tensile strain in Te nanowire.
- Intriguing and tunable optical properties of 1D Te nanowire by strain engineering.

## Graphical abstract

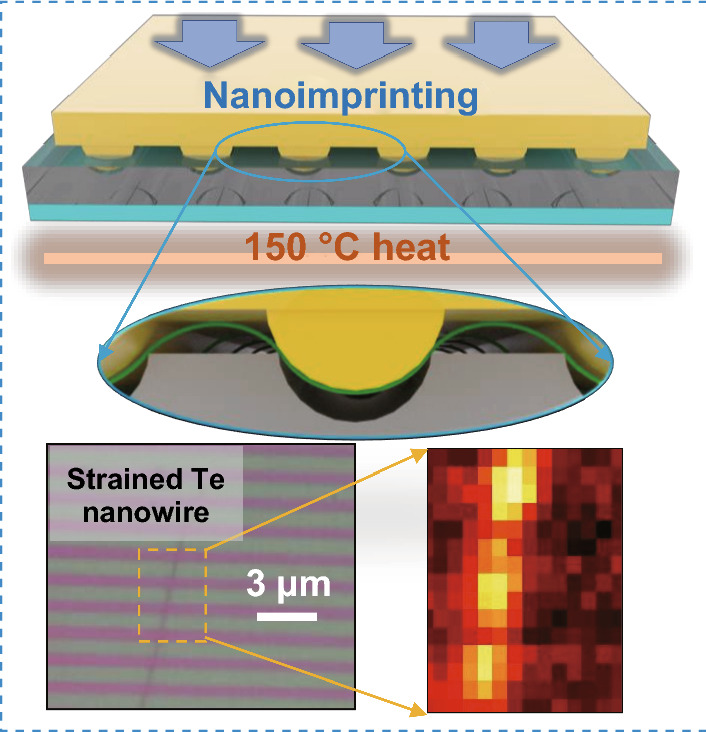

## Introduction

The low-dimensional, highly anisotropic geometries, and superior mechanical properties of one-dimensional (1D) nanomaterials [1–3] allow the introduction of enormous elastic strains [3–7] (e.g., > 1%) and even designer strain fields [8–11] without fracture. These materials are amenable to exquisite mechanical engineering [9, 12, 13] with a wide range of tunability inaccessible to bulk or thin-film materials. Such engineering capability offers unprecedented possibilities for probing intriguing physics and materials science in the 1D limit [14–17]. The strong coupling of mechanical strain to various internal degrees of freedom involving charges, photons, spins, etc., also enables the design and implementation of device technologies with novel functionalities, such as stretchable electronics/optoelectronics [18–21], quantum straintronics [22–25], electromechanical sensors [26–28], strain-engineered piezotronics [29–32], and mechanically enhanced catalysis [33–35]. Strain engineering in 1D semiconductors such as silicon [6, 7, 36] and ZnO [29, 31, 37] nanowires has been explored theoretically [38–44] and experimentally [7, 36, 45, 46] as an effective approach to rationally engineer the crystal structure, semiconductor properties, and device functions of the related materials [47–54].

Several techniques have been demonstrated to be feasible for introducing controlled strains in 1D materials through, e.g., bending of the flexible substrates [55], elongating of the elastic substrates [56], thermally induced expansion of the substrates [57, 58], and embossing/imprinting the active materials to patterned substrates [59–62]. Among these strategies, imprinting the 1D nanowires into designed patterns attracts increased attention due to its capability to parallelly form the different segments of the materials into wrinkled structures with controlled periodicities, amplitudes, and orientations, closely following the predefined patterns on the host substrates. Compared to other nanopatterning schemes based on lithography techniques, such as photolithography [63], electron beam lithography (EBL) [64], focused ion beam lithography (FIBL) [65], nanoimprinting exhibits unique advantages for resist-free, high-resolution, low-cost, rapid, and high-throughput patterning [66–71]. Moreover, nanoimprinting can also introduce elastic strains in designed patterns with nanoscale resolution [66, 72, 73]. Nevertheless, much less was known for the application of nanoimprinting on the parallel forming of semiconductor nanowires.

As a Group VI element, bulk tellurium (Te) is a *p*-type semiconductor with a narrow bandgap of 0.35 eV [74, 75]. Te exhibits interesting properties such as semiconducting [76, 77], thermoelectric [78], piezoelectric [79, 80] for application in electronics, energy devices, and sensors [81, 82]. Te’s intriguing trigonal crystal lattice [76, 77, 82, 83] consists of anisotropic 1D chiral chains. Each Te atom is covalently bonded with its two nearest neighbors on the same chain, and the interchain interaction is weaker than the covalent bond [74]. A systematic study on strain engineering the 1D Te’s anisotropic properties will be helpful for providing fundamental insights of the coupling between mechanical strains and various internal degrees of freedom in Te nanomaterials [76, 77, 80] and other materials sharing similar 1D chain structures [84–86], as well as enabling the design and development of novel smart devices capable of actively interacting with the working environment [31, 87, 88].

Here, we systematically investigated the strain-engineered anisotropic optical properties in 1D Te nanowires, through designing and introducing a controlled strain field in solution-grown ultralong Te nanowires, using a resist-free thermally assisted nanoimprinting method. The magnitude of induced strains can be tuned by adjusting the imprinting pressure, the nanowire diameter, and the patterns on the substrates. The observed Raman spectra from the chiral-chain lattice of 1D Te, dependent on the magnitude of the introduced strain, reveal the strong lattice vibration response under the corresponding strain conditions. Our results suggest the potential of 1D Te as a promising candidate for enabling flexible electronics [89, 90], deformable optoelectronics [91, 92], and wearable sensors [26, 28, 31, 93]. The experimental platform can also enable the exquisite mechanical control in 1D Te and other 1D nanomaterials with substrate-induced, on-demand, and controlled strains.

## Results and Discussion

**Fig. 1:**
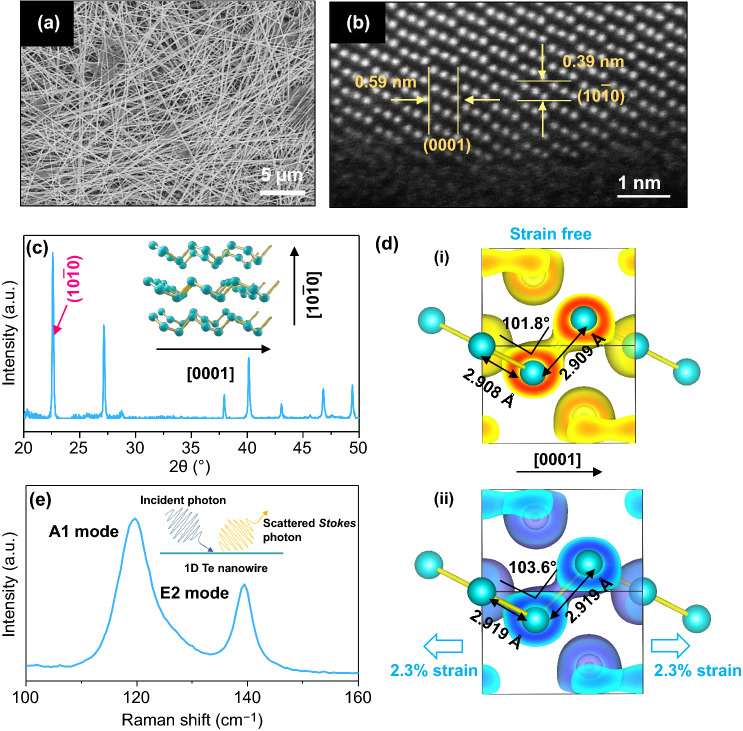
Characterization of 1D Te nanowire. **a** Scanning electron microscopy (SEM) image of the as-synthesized 1D Te nanowire. **b** High-resolution transmission electron microscope image of the 1D Te nanowire at the edge. **c** XRD results of the 1D Te nanowire. **d** Atomic structures of the Te crystals without and with tensile strain. The isosurface value for electron density is 0.03 *e*/Bohr^3^, **e** Raman spectra of the 1D Te nanowire

Figure 1a shows the morphology of the solution synthesized ultralong Te nanowires (Methods) with lengths over hundreds of micrometers. The high-resolution transmission electron microscope (HRTEM) (Fig. 1b) characterization for the side edge of a Te nanowire reveals the atomically resolved lattice of Te nanowire. In Fig. 1b, the measured lattice spacings of 5.9 and 3.9 Å correspond to the (0001) and (10$\bar{1}$0) planes for trigonal tellurium, respectively, which matches well with the previous reports [94, 95]. The HRTEM results also suggest the crystal orientation for the nanowires’ length directions is along [0001]. X-ray powder diffraction (XRD) results of the powdered Te nanowires sample (Fig. 1c) further verify that the product consists of crystalline elemental tellurium. All the diffraction peaks can be indexed as the trigonal tellurium phase (JCPDS No. 36-1452) [94, 96]. The high relative intensity of (10$\bar{1}$0) diffraction peaks indicates the top surface of 1D Te nanowires are dominated by the (10$\bar{1}$0) planes (see the atomic structure in Fig. 1c insert). It should be noted that the helical chains of Te atoms (hence a threefold screw symmetry) are packed along the longitudinal direction of Te nanowires ([0001] direction), and the radial stacking in the Te nanowire is along the [10$\bar{1}$0] direction. We performed density functional theory (DFT) calculations to explore the difference in the optimized structures without and with tensile strains (see Methods). As is shown in Fig. 1d(i), the bond distances between Te and its nearest Te atoms along the [0001] chain direction are 2.909 Å, and the angle between the two adjacent bonds is 101.9°. After 2.3% tensile strain was applied along the [0001] direction, the bond distances and angle change to 2.919 Å, 2.918 Å, and 103.5°, respectively. In Fig. 1d(ii), we also marked the electron density with 0% (yellow) and 2.3% (blue) tensile strain. Such structural change could lead to a significant change in the lattice vibration. It is well understood that Raman spectroscopy can sensitively reveal the surface vibration of the sample (Fig. 1e insert). Figure 1e shows the Raman spectra of a Te nanowire sample with a diameter larger than 40 nm. The Raman signal was excited by a 633-nm laser along the [10$\bar{1}$0] direction of Te nanowires. Two Raman modes locating at 121 cm^−1^ and 143 cm^−1^ can be identified, which agree well with the previous results for bulk tellurium [76, 97]. Further detailed exploration for strain-engineered Raman spectra of 1D Te will be discussed in later sections.

**Fig. 2:**
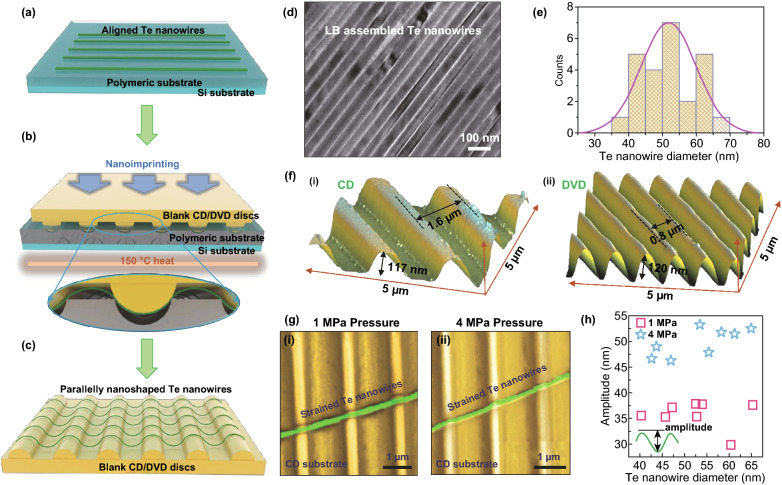
Fabrication and topography characterization of the parallelly deformed 1D Te. **a**–**c** Schematic procedure of fabricating the wavy geometry of 1D Te nanowire on optical disks. **d** Transmission electron microscope image of the assembled 1D Te nanowire on the copper grid. **e** The thickness distribution of the as-synthesized 1D Te nanowire. **f** Atomic force microscopes (AFM) image of the optical disks. **g** Scanning electron microscope images of the deformed 1D Te nanowire by 1 and 4 MPa nanoimprinting pressure. **h** The relationship between the deformed amplitude and Te nanowire diameter

A schematic configuration illustrating the process scheme for nanoimprinting induced parallel forming of Te nanowires is shown in Figs. 2a–c. To minimize the undesired entanglement and agglomeration (Fig. 1a) of the as-synthesized ultralong, thin Te nanowires for ensuring a controlled straining process, we first performed a Langmuir–Blodgett (LB) process [98, 99] to transfer and assemble the Te nanowires from the synthesis solution into a monolayer assembly [100] on a soft polyester terephthalate (PET) substrate. Figure 2d shows a TEM image of the LB-assembled Te nanowires on a copper grid. Furthermore, such an LB assembly process facilitates the orientation alignment of the constituent nanowires in a large-scale assembly [101] (Fig. 2a, d). The statistic histogram in Fig. 2e shows that the as-synthesized Te nanowires possess diameters from 35 to 70 nm. Through tuning the pressure of imprinting (method), the aligned Te nanowires can be stamped onto mold substrates with periodic gratings (blank CD or DVD disk), which induces a periodic strain field in the Te nanowire (Fig. 2c). The magnitude of the strain can be engineered by adjusting the pressure power, the diameter of the nanowire, and using CD or DVD substrates. Figure 2b illustrates the nanoimprint-induced straining process. The resist-free thermal nanoimprinting process is a pattern emboss method. When the mold indents the PET at relatively low pressure (e.g., 1 MPa) with heat, the plastic flow of the PET substrate allows the mold to move inwards the PET, which induces a controllable local strain in the segment of Te nanowire sandwiched in between. The selection of suitable molding substrates with patterned surfaces is crucial for this method [59]. The commercial compact disk (CD) and digital versatile disk (DVD) encode their information by a spiral track molded onto the top of a polycarbonate layer [102]. The spiral tracks on blank CDs and DVDs have depths around hundreds of nanometers, and the distance between each neighboring track varies from 850 nm to 2 um [102]. The surface patterns with precise periodicities and low cost of blank CD and DVD make them ideal substrates for the nanoimprinting process. The nanoimprint strained Te nanowires, supported on the CD/DVD substrate, is expected to exhibit a sinusoidal shape where the wavelength of the strain field in Te nanowire equals to the distance of the neighboring indentations in the CD or DVD substrates (Fig. 2c), and the amplitude of the strain field is primarily determined by the pressure of the nanoimprinting process [67, 69, 71].

Atomic force microscope (AFM) was used to characterize the topography (in terms of the periodicity and the depth of the mold) of the starting blank CD [Fig. 2f(i)] and DVD substrates [Fig. 2f(ii)]. The results show that the grating depth and periodicity are 117 nm and 1.6 µm for the CD and 120 nm and 0.8 µm for the DVD molds, respectively. The scanning electron microscopy (SEM) images in Fig. 2g illustrate a Te nanowire (diameter ~ 50 nm), which is parallelly formed on a CD substrate with different nanoimprinting pressures. We optimized the process temperature at 150 °C and kept this value for all nanoimprinting experiments in this work. We used two nanoimprinting pressures (1 and 4 MPa) in our experiment. As is shown in the false-color SEM images in Fig. 2g, the bright gold areas are the top surfaces of the indentations on the CD mold, and the dark gold areas are the valley areas between these indentations. We did not observe cracking or fracture in the Te nanowire for either the low-pressure (1 MPa) or the high-pressure (4 MPa) nanoimprinting process, which indicates the superior mechanical property of crystalline Te nanowire [79, 84]. Figure 2h summarizes the measured deformation amplitudes of the sinusoidal shape 1D Te nanowire by AFM with different nanoimprinting pressures. We examined 8 nanowires with different diameters for each pressure. Under 1-MPa pressure, the amplitude ranges from 30 to 38 nm, while the amplitude reaches to 45–55 nm with the 4-MPa pressure imprinting.

**Fig. 3:**
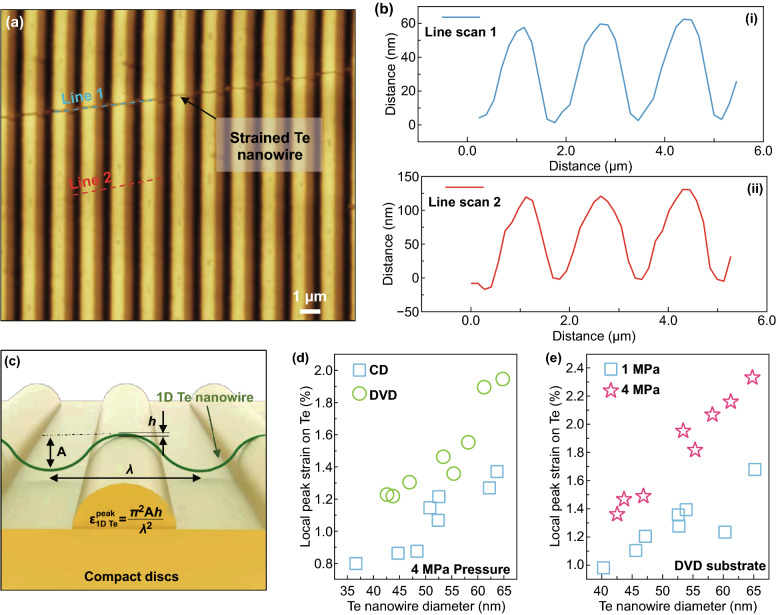
Analysis of the deformed 1D Te on the optical disks. **a** Atomic force microscopes (AFM) image of the strained 1D Te nanowire on compact disks. **b** Two line-scan results from **a**. **c** The strain analysis of the deformed 1D Te nanowire on the compact disks. The relationship between the local peak strain and Te nanowire diameter **d** on different disks and **e** different nanoimprinting pressure

We also characterized the topological configuration of strained Te nanowires (diameter ~ 45 nm) on a CD mold (Fig. 3). The AFM line scans performed in the regions for the Te nanowire and the CD substrate (Fig. 3a) show the periodic sinusoidal shapes for both the strained Te nanowire and the CD substrate [Fig. 3b(i), (ii)]. The amplitude and wavelength for the sinusoidal shape Te nanowire (line 1) were determined to be 50 nm and 1.6 µm under 4 MPa. The amplitude and wavelength for the CD patterns (line 2) were determined to be 120 nm and 1.6 µm, respectively. The bending mechanics in the deformed Te nanowire can be estimated using the schematics shown in Fig. 3c, where a nanowire with a diameter of $h$ is bent to a sinusoidal curvature. The nanowire segment on top of the CD indentation is tensilely strained [103, 104]. In the regime where $h$ is small compared to the radius of the curvature *r* ($h$ = 40–70 nm and *r* = 1–2.5 µm), the peak strain induced in the nanowire can be estimated by the equation:

1 $$ \varepsilon_{{ 1 {\text{D Te}}}}^{\text{peak}} = \frac{h}{2r} $$

Here, *r* is the radius of curvature at the peak of the wavy, which is generally expressed by

2 $$ r = - \left. {\frac{1}{y''}} \right|_{{x = \pm \left[ {\frac{(2n - 1)\pi }{2k}} \right]}} $$

where *n* is an integer and *y*″ is the second derivative of *y* with respect to *x*. The shape of the wavy 1D Te can be expressed by a sine function,

3 $$ y = \left( {A/2} \right) \cdot \sin \left( {kx} \right) \, (k = 2\pi /\lambda ) $$

where A and $\lambda$ are amplitude and wavelength of the sinusoidal Te nanowire (Fig. 3c). As a result, the peak strain induced in the Te nanowire can be expressed as

4 $$ \varepsilon_{{ 1 {\text{D Te}}}}^{\text{peak}} = \frac{{\pi^{2} Ah}}{{\lambda^{2} }} $$

where the amplitude *A*, diameter $h$, and wavelength $\lambda$ can all be determined from the AFM results (Fig. 3b). Figure 3 d, e summarizes the derived local peak strains for the deformed Te nanowires on CD and DVD substrates with different imprinting pressures. In Fig. 3d, we examined the local strains of 16 different Te nanowires with diameters ranging from 35 to 65 nm on CD and DVD molds, when we maintained a 4 MPa pressure for the nanoimprinting. The green circles represent the 8 Te nanowires on the CD substrates, and the blue squares represent the 8 Te nanowires on the DVD substrates. The local peak strains in Te nanowires increase with the increased nanowire diameters. For instance, the local peak strain is 0.8% for Te nanowire with a 36.6 nm diameter and increases to 1.9% for Te nanowire with a 64.8 nm diameter. Moreover, our results revealed that the use of DVD substrates could induce more significant local strains since the wavelength $\lambda$ of the deformed Te nanowires on DVD substrates is smaller than on CD substrates. Figure 3e summarizes the results when two imprinting processes (1-MPa and 4-MPa) were applied to Te nanowires (diameters ranging from 40 to 65 nm) on DVD substrates. The local peak strains increase with increased nanowire diameters. Also, a higher imprinting pressure (i.e., 4 MPa) will result in more significant local strains in the nanowires. For example, the local peak strain in a Te nanowire (diameter ~ 65 nm) is 1.7% when 1 MPa pressure process was applied, and reaches to 2.3% when 4 MPa pressure was applied. Consequently, we could rationally tune the local strains in the formed Te nanowires through manipulating the nanowire diameter, mold type (CD or DVD), and the imprinting pressure.

**Fig. 4:**
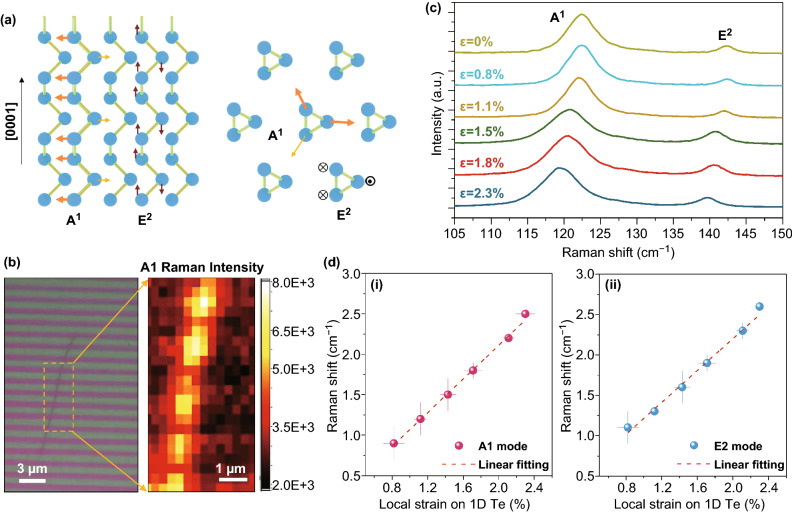
Raman spectroscopy results of the strained 1D Te nanowire. **a** Schematic shows the main Raman-active modes in Te crystal. **b** Optical image and Raman intensity mapping of the deformed 1D Te nanowire. **c** Raman spectra of the deformed 1D with different local strain. **d** Quantitative analysis of the Raman frequency shift of the deformed 1D Te with different local strains. The average values from 3 technical replicates are indicated, and error bars represent one standard deviation for each set of replicates

These parallelly nanoshaped tellurium nanowires with controlled local strains provide an ideal system to explore the strain-engineered optical property at the nanoscale. To this end, we characterized the strained 1D Te nanowires with Raman spectroscopy at room temperature. Figure 4a illustrates the two vibration modes in the chiral-chain structure of Te. The *A*^1^ mode represents the breathing vibration in the (10$\bar{1}$0) plane, and *E*^2^ mode is the asymmetric stretching along [0001] direction in tellurium [76, 97] (Fig. 4a). We first performed Raman spectra mapping for the area enclosed by the orange dashed lines in the optical image in Fig. 4b. We found a periodic distribution of the Raman peak intensity of *A*^1^ mode spanning across the entire nanowire, which is consistent with the topography variation in the strained nanowire (Fig. 4b). This result not only confirmed the periodic deformations in the strained nanowire but also helped us identify the regions with the local peak strains with nanoscale resolution. Subsequently, we examined the strain-induced shift in the Raman peaks from the positions where the local segments of the nanowire experienced the peak strains (bright spots in the Raman mapping in Fig. 4b). Figure 4c shows the strain-engineered Raman shifts in the *A*^1^ and *E*^2^ modes when the local peak strain was increased from 0 to 2.3%. When the Te nanowire (diameter ~ 40 nm) was naturally deposited on the substrate without introducing the nanoimprinting strain, two Raman-active modes locating at 121 cm^−1^ (*A*^1^-mode) and 143 cm^−1^ (*E*^2^-mode) were identified, which is consistent with previous literature and our results in Fig. 1. When we increased the local peak strains, both modes showed a significant red shift in the Raman peaks (Fig. 4c). The *A*^1^-mode peak shifted to 117.6 cm^−1,^ and the *E*^2^-mode peak shifted to 140.3 cm^−1^ when the strain was increased to 2.3%. We plotted the Raman frequency shift as the function of the local strains (Fig. 4d), and the results revealed a linear trend. The linear relationship between the Raman frequency shift and the uniaxial tensile strain *ε* is given by Δ*ω* = *γε* [105, 106] where *γ* is the phonon deformation potential coefficient, *ε* is the uniaxial tensile strain on the materials, and Δ*ω* is the phonon frequency shift. The rate of the shift is determined to be approximately 1.1 cm^−1^ %^−1^ for *A*^1^-mode [Fig. 4d(i)] and 1.2 cm^−1^ %^−1^ for *E*^2^-mode [Fig. 4d(ii)], respectively. These results are also consistent with our previous results for 2D Te [107]. The response of Raman spectra with different strains can be explained by examining the intrinsic vibration modes of Te [97] and the electro-optic interactions in Te [106]. When the tensile strain was applied along the [0001] direction of the Te nanowire, the length for the Te–Te covalent bond was elongated (Fig. 1e), which weakens the interatomic interaction [107, 108]. As a result, the stretching along the [0001] helical chain direction became softening, leading to a decrease in the vibration frequency of *E*^2^-mode [109, 110]. Meanwhile, this softening effect leads to the weaker Te atom vibration in (10$\bar{1}$0) plane, resulting in a decreased vibration frequency of Te helical chain breathing in this plane, which is associated with the observed redshift in *A*^1^-mode peak.

## Conclusions

In summary, we systematically investigated the strain-engineered optical properties in 1D chiral semiconductor tellurium, through applying a facile resist-free low-temperature nanoimprinting strategy to induce parallel nanoshaping in solution-grown ultralong 1D Te nanowires using commercial CD and DVD substrates. The observed Raman spectra from the chiral-chain lattice of 1D Te reveal the anisotropic lattice vibration under the corresponding straining conditions. We performed synergistic efforts combining theoretical and experimental investigations to elucidate the impacts of strain engineering on the lattice structure and optical properties of the 1D helical chain tellurium. The nanoimprinting-introduced elastic strain in Te nanowires is self-sustained and tunable, which could be further utilized for modulating the electrical, electrical–optical, mechanical, and other material properties of Te nanowires. The exquisite strain engineering on 1D Te could open up opportunities in flexible, wearable integrated electronic systems, as well as promising elastic strain engineering that takes advantage of the drastically changed electronic and optical properties under large lattice strain. The experimental platform can also enable the exquisite mechanical control in 1D Te and other nanomaterials with substrate-induced, on-demand, and controlled strains.

## Materials and Methods

### Materials

PVP, hydrazine hydrate (85%, w/w), acetone, ethylene glycol (EG), aqueous ammonia solution (25–28%, w/w), *N*,*N*-dimethylformamide (DMF), CHCl_3_ were purchased from Sigma-Aldrich. Na_2_TeO_3_ (97%) was purchased from Alfa Aesar. All chemical reagents were used without further purification. Double-distilled deionized water (DIW; 18.2 megaohms) was used for the synthesis.

### Synthesis of Te Nanowires

In the typical synthesis, 0.1 g Na_2_TeO_3_ and 1 g PVP were put into 15 mL double-distilled water and 15 mL EG at room temperature under magnetic stirring to form a homogeneous solution. The resulting solution was poured into a 50-mL Teflon-lined steel autoclave, which was then filled with aqueous ammonia solution and hydrazine hydrate. The autoclave was sealed and maintained at 180 °C for 3 h. Then, the autoclave was cooled to room temperature naturally. The resulting blue products were precipitated by centrifuge at 5000 rpm for 5 min and washed with distilled water and acetone (to remove any ions remaining in the final product).

### Langmuir–Blodgett (LB) Transfer of 1D Te

The hydrophilic Te nanowires can be transferred to substrates by the Langmuir–Blodgett (LB) technique. The washed Te nanowires were mixed with a certain volume ratio of *N*,*N*-dimethylformamide (DMF), and CHCl_3_. Then, the above solution was dropped into the deionized water. Then, Te nanowire monolayer was prepared at 25 °C using an LB trough (Nima Technology, 312D). The trough was filled with Millipore Milli-Q water (resistivity of 18.2 Mohm cm) until it brimmed just over the top by about 2 mm. Five milliliters of the solution of Te nanowires were precipitated by adding 12 mL of acetone and centrifuging at 5000 rpm for 5 min. After 30 min, the monolayer Te nanowire can be transferred to any substrates. The monolayer was lifted at a pressure of 23 mN m^−1^, and the deposition speed was maintained at 4 mm min^−1^. The centimeter-scale assembly of nanowires can be realized with controlled density, orientation, and spacing [99, 100, 111]. The floated nanowires can be parallelly aligned by a controlled barrier.

### Nanoimprinting Process

Before nanoimprinting, the polyester terephthalate thin film was spin-coated on a silicon wafer with 4000 rpm in 45 s. The commercial blank compact disks were sonicated in acetone and water. The nanoimprinting was performed using Nanonex NX 2000.

### DFT Calculations

Vienna Ab initio Simulation Package (VASP) 5.4.4 [112, 113] was used for performing DFT calculations with the projector augmented wave method [114]. The exchange–correlation functional of OptPBE-vdW [115] was used to take into account van der Waals interactions. The plane-wave basis set cutoff was 400 eV, and a Gamma-centered k-point mesh was set to 5 × 5 × 3. Gaussian smearing of 0.1 eV was used. The electronic wavefunction convergence threshold was 10^−8^ eV, and a 0.01 Ev Å^−1^ convergence criterion for forces on each atom was adopted.

### Characterization

The thickness and morphology of the substrate surface were determined by AFM (Keysight 5500). High-resolution STEM/TEM imaging and SAED have been performed using a probe-corrected JEM-ARM 200F (JEOL USA, Inc.) operated at 200 kV, and EDS has been collected by an X-MaxN100TLE detector (Oxford Instruments). A field emission scanning electron microscope (Hitachi S-4800 Field Emission SEM) was used to characterize the morphologies of Te nanowires.

## References

[1] Wu B, Heidelberg A, Boland JJ (2005). Mechanical properties of ultrahigh-strength gold nanowires. Nat. Mater..

[2] Barnett RN, Landman U (1997). Cluster-derived structures and conductance fluctuations in nanowires. Nature.

[3] Wong EW, Sheehan PE, Lieber CM (1997). Nanobeam mechanics: elasticity, strength, and toughness of nanorods and nanotubes. Science.

[4] Herring C, Galt JK (1952). Elastic and plastic properties of very small metal specimens. Phys. Rev..

[5] Song J, Wang X, Riedo E, Wang ZL (2005). Elastic property of vertically aligned nanowires. Nano Lett..

[6] Gordon MJ, Baron T, Dhalluin F, Gentile P, Ferret P (2009). Size effects in mechanical deformation and fracture of cantilevered silicon nanowires. Nano Lett..

[7] Minamisawa RA, Süess MJ, Spolenak R, Faist J, David C, Gobrecht J, Bourdelle KK, Sigg H (2012). Top-down fabricated silicon nanowires under tensile elastic strain up to 4.5%. Nat. Commun..

[8] Larsson MW, Wagner JB, Wallin M, Håkansson P, Fröberg LE, Samuelson L, Wallenberg LR (2006). Strain mapping in free-standing heterostructured wurtzite InAs/InP nanowires. Nanotechnology.

[9] Adelung R, Aktas OC, Franc J, Biswas A, Kunz R (2004). Strain-controlled growth of nanowires within thin-film cracks. Nat. Mater..

[10] Nakamura A, Matsunaga K, Tohma J, Yamamoto T, Ikuhara Y (2003). Conducting nanowires in insulating ceramics. Nat. Mater..

[11] Cheng G, Miao C, Qin Q, Li J, Xu F (2015). Large anelasticity and associated energy dissipation in single-crystalline nanowires. Nat. Nanotechnol..

[12] Qin Q, Yin S, Cheng G, Li X, Chang T-H (2015). Recoverable plasticity in penta-twinned metallic nanowires governed by dislocation nucleation and retraction. Nat. Commun..

[13] Yu G, Cao A, Lieber CM (2007). Large-area blown bubble films of aligned nanowires and carbon nanotubes. Nat. Nanotechnol..

[14] Goldthorpe IA, Marshall AF, McIntyre PC (2008). Synthesis and strain relaxation of Ge-core/Si-shell nanowire arrays. Nano Lett..

[15] Signorello G, Lörtscher E, Khomyakov PA, Karg S, Dheeraj DL (2014). Inducing a direct-to-pseudodirect bandgap transition in wurtzite GaAs nanowires with uniaxial stress. Nat. Commun..

[16] Diao J, Gall K, Dunn ML (2003). Surface-stress-induced phase transformation in metal nanowires. Nat. Mater..

[17] Johansson J, Karlsson LS, Patrik C, Svensson T, Mårtensson T, Wacaser BA (2006). Structural properties of 〈111〉B-oriented III–V nanowires. Nat. Mater..

[18] Ferry DK (2008). Nanowires in nanoelectronics. Science.

[19] Tomioka K, Yoshimura M, Fukui T (2012). A III–V nanowire channel on silicon for high-performance vertical transistors. Nature.

[20] Malvankar NS, Vargas M, Nevin KP, Franks AE, Leang C (2011). Tunable metallic-like conductivity in microbial nanowire networks. Nat. Nanotechnol..

[21] Feng C, Wang S, Yin L, Li X, Yao M (2018). Significant strain-induced orbital reconstruction and strong interfacial magnetism in TiNi(Nb)/ferromagnet/oxide heterostructures via oxygen manipulation. Adv. Funct. Mater..

[22] Kremer PE, Dada AC, Kumar P, Ma Y, Kumar S, Clarke E, Gerardot BD (2014). Strain-tunable quantum dot embedded in a nanowire antenna. Phys. Rev. B.

[23] Krogstrup P, Ziino NLB, Chang W, Albrecht SM, Madsen MH (2015). Epitaxy of semiconductor–superconductor nanowires. Nat. Mater..

[24] Smogunov A, Dal Corso A, Delin A, Weht R, Tosatti E (2008). Colossal magnetic anisotropy of monatomic free and deposited platinum nanowires. Nat. Nanotechnol..

[25] Bezryadin A, Lau CN, Tinkham M (2000). Quantum suppression of superconductivity in ultrathin nanowires. Nature.

[26] Takei K, Takahashi T, Ho JC, Ko H, Gillies AG (2010). Nanowire active-matrix circuitry for low-voltage macroscale artificial skin. Nat. Mater..

[27] Xu S, Hansen BJ, Wang ZL (2010). Piezoelectric-nanowire-enabled power source for driving wireless microelectronics. Nat. Commun..

[28] Zheng G, Patolsky F, Cui Y, Wang WU, Lieber CM (2005). Multiplexed electrical detection of cancer markers with nanowire sensor arrays. Nat. Biotechnol..

[29] Wang ZL, Song J (2006). Piezoelectric nanogenerators based on zinc oxide nanowire arrays. Science.

[30] Wu W, Wei Y, Wang ZL (2010). Strain-gated piezotronic logic nanodevices. Adv. Mater..

[31] Wu W, Wen X, Wang ZL (2013). Taxel-addressable matrix of vertical-nanowire piezotronic transistors for active and adaptive tactile imaging. Science.

[32] Wu W, Wang ZL (2011). Piezotronic nanowire-based resistive switches as programmable electromechanical memories. Nano Lett..

[33] Ling T, Yan D-Y, Wang H, Jiao Y, Hu Z (2017). Activating cobalt(II) oxide nanorods for efficient electrocatalysis by strain engineering. Nat. Commun..

[34] Luo M, Guo S (2017). Strain-controlled electrocatalysis on multimetallic nanomaterials. Nat. Rev. Mater..

[35] Bu L, Guo S, Zhang X, Shen X, Su D (2016). Surface engineering of hierarchical platinum-cobalt nanowires for efficient electrocatalysis. Nat. Commun..

[36] He R, Yang P (2006). Giant piezoresistance effect in silicon nanowires. Nat. Nanotechnol..

[37] Wu W, Pan C, Zhang Y, Wen X, Wang ZL (2013). Piezotronics and piezo-phototronics—from single nanodevices to array of devices and then to integrated functional system. Nano Today.

[38] Yue Y, Liu P, Zhang Z, Han X, Ma E (2011). Approaching the theoretical elastic strain limit in copper nanowires. Nano Lett..

[39] Marini C, Chermisi D, Lavagnini M, Di Castro D, Petrillo C (2012). High-pressure phases of crystalline tellurium: a combined Raman and ab initio study. Phys. Rev. B.

[40] Shiri D, Kong Y, Buin A, Anantram MP (2008). Strain induced change of bandgap and effective mass in silicon nanowires. Appl. Phys. Lett..

[41] Park HS (2012). Surface stress effects on the critical buckling strains of silicon nanowires. Comput. Mater. Sci..

[42] Salazar F, Trejo-Baños A, Miranda A, Pérez LA, Cruz-Irisson M (2019). Interstitial sodium and lithium doping effects on the electronic and mechanical properties of silicon nanowires: a DFT study. J. Mol. Model..

[43] Esfahani MN (2019). Surface stress effects on the mechanical properties of silicon nanowires: a molecular dynamics simulation. J. Appl. Phys..

[44] Zhang H, Fung K-Y, Zhuang Y, Cao K, Song J, Hu A, Lu Y (2019). Fracture of a silicon nanowire at ultra-large elastic strain. Acta Mech..

[45] Wölz M, Ramsteiner M, Kaganer VM, Brandt O, Geelhaar L, Riechert H (2013). Strain engineering of nanowire multi-quantum well demonstrated by raman spectroscopy. Nano Lett..

[46] Treacy MMJ, Ebbesen TW, Gibson JM (1996). Exceptionally high Young’s modulus observed for individual carbon nanotubes. Nature.

[47] Michael Cai W, Juyoung L, Pilgyu K, Jonghyun C, Peter K, Keong Y, SungWoo N (1996). 2D Mater..

[48] Jiang H, Khang D-Y, Song J, Sun Y, Huang Y, Rogers JA (2007). Finite deformation mechanics in buckled thin films on compliant supports. Proc. Natl. Acad. Sci..

[49] Deng S, Sumant AV, Berry V (2018). Strain engineering in two-dimensional nanomaterials beyond graphene. Nano Today.

[50] Rafael R, Andrés C-G, Emmanuele C, Francisco G (2015). Strain engineering in semiconducting two-dimensional crystals. J. Phys.: Condens. Matter.

[51] Khang DY, Rogers JA, Lee HH (2009). Mechanical buckling: mechanics, metrology, and stretchable electronics. Adv. Funct. Mater..

[52] Koo WH, Jeong SM, Araoka F, Ishikawa K, Nishimura S, Toyooka T, Takezoe H (2010). Light extraction from organic light-emitting diodes enhanced by spontaneously formed buckles. Nat. Photonics.

[53] Feng C, Li Y, Wang L, Cao Y, Yao M (2020). Giant strain control of antiferromagnetic moment in metallic FeMn by tuning exchange spring structure. Adv. Funct. Mater..

[54] Wang L, Feng C, Li Y, Meng F, Wang S (2019). Switchable magnetic anisotropy of ferromagnets by dual-ion-manipulated orbital engineering. ACS Appl. Mater. Interfaces..

[55] Mohiuddin TMG, Lombardo A, Nair RR, Bonetti A, Savini G (2009). Uniaxial strain in graphene by Raman spectroscopy: G peak splitting, Grüneisen parameters, and sample orientation. Phys. Rev. B.

[56] Ryu SY, Xiao J, Park WI, Son KS, Huang YY, Paik U, Rogers JA (2009). Lateral buckling mechanics in silicon nanowires on elastomeric substrates. Nano Lett..

[57] Gerd P, Andres C-G, Michele B, Herre SJVDZ, Gary AS, Agnieszka K, Thomas H, Christian S, Tobias K (2015). 2D Mater..

[58] Bowden N, Brittain S, Evans AG, Hutchinson JW, Whitesides GM (1998). Spontaneous formation of ordered structures in thin films of metals supported on an elastomeric polymer. Nature.

[59] Li H, Contryman AW, Qian X, Ardakani SM, Gong Y (2015). Correction: Corrigendum: Optoelectronic crystal of artificial atoms in strain-textured molybdenum disulphide. Nat. Commun..

[60] Xia Y, Whitesides GM (1998). Soft lithography. Angew. Chem. Int. Ed..

[61] Jin S, Wang Y, Motlag M, Gao S, Xu J, Nian Q, Wu W, Cheng GJ (2018). Large-area direct laser-shock imprinting of a 3D biomimic hierarchical metal surface for triboelectric nanogenerators. Adv. Mater..

[62] Jin S, Zhou Z, Sakr ESA, Motlag M, Huang X (2019). Scalable nanoshaping of hierarchical metallic patterns with multiplex laser shock imprinting using soft optical disks. Small.

[63] Hölz K, Schaudy E, Lietard J, Somoza MM (2019). Multi-level patterning nucleic acid photolithography. Nat. Commun..

[64] Ito T, Okazaki S (2000). Pushing the limits of lithography. Nature.

[65] Horák M, Bukvišová K, Švarc V, Jaskowiec J, Křápek V, Šikola T (2018). Comparative study of plasmonic antennas fabricated by electron beam and focused ion beam lithography. Sci. Rep..

[66] Chou SY, Krauss PR, Renstrom PJ (1996). Imprint lithography with 25-nanometer resolution. Science.

[67] Guo LJ (2007). Nanoimprint lithography: methods and material requirements. Adv. Mater..

[68] Guo LJ (2004). Recent progress in nanoimprint technology and its applications. J. Phys. D-Appl. Phys..

[69] Chou SY, Keimel C, Gu J (2002). Ultrafast and direct imprint of nanostructures in silicon. Nature.

[70] Krauss PR, Chou SY (1997). Nano-compact disks with 400 Gbit/in2400 Gbit/in2 storage density fabricated using nanoimprint lithography and read with proximal probe. Appl. Phys. Lett..

[71] Varghese LT, Fan L, Xuan Y, Tansarawiput C, Kim S, Qi M (2013). Resistless nanoimprinting in metal for plasmonic nanostructures. Small.

[72] Li Z, Gu Y, Wang L, Ge H, Wu W (2009). Hybrid nanoimprint–soft lithography with sub-15 nm resolution. Nano Lett..

[73] Chou SY, Krauss PR (1997). Imprint lithography with sub-10 nm feature size and high throughput. Microelectron. Eng..

[74] von Hippel A (1948). Structure and conductivity in the VIb group of the periodic system. J. Chem. Phys..

[75] Arlt G, Quadflieg P (1969). Electronic displacement in tellurium by mechanical strain. Phys. Status Solidi (B).

[76] Wang Y, Qiu G, Wang R, Huang S, Wang Q (2018). Field-effect transistors made from solution-grown two-dimensional tellurene. Nat. Electron..

[77] Wang Y, de Souza Borges Ferreira R, Wang R, Qiu G, Li G (2019). Data-driven and probabilistic learning of the process–structure–property relationship in solution-grown tellurene for optimized nanomanufacturing of high-performance nanoelectronics. Nano Energy.

[78] Lin S, Li W, Chen Z, Shen J, Ge B, Pei Y (2016). Tellurium as a high-performance elemental thermoelectric. Nat. Commun..

[79] Lee TI, Lee S, Lee E, Sohn S, Lee Y (2013). High-power density piezoelectric energy harvesting using radially strained ultrathin trigonal tellurium nanowire assembly. Adv. Mater..

[80] Wang Y, Wang R, Wan S, Wang Q, Kim MJ, Ding D, Wu W (2019). Scalable nanomanufacturing and assembly of chiral-chain piezoelectric tellurium nanowires for wearable self-powered cardiovascular monitoring. Nano Futures.

[81] Gao S, Wang Y, Wang R, Wu W (2017). Piezotronic effect in 1D van der Waals solid of elemental tellurium nanobelt for smart adaptive electronics. Semicond. Sci. Technol..

[82] Wu W, Qiu G, Wang Y, Wang R, Ye P (2018). Tellurene: its physical properties,
scalable nanomanufacturing, and device applications. Chem. Soc. Rev..

[83] Ibers J (2009). Tellurium in a twist. Nat. Chem..

[84] Wu M, Wang Y, Gao S, Wang R (2019). Solution-synthesized chiral piezoelectric selenium nanowires for wearable self-powered human-integrated monitoring. Nano Energy.

[85] Ben-Moshe A, Govorov AO, Markovich G (2013). Enantioselective synthesis of intrinsically chiral mercury sulfide nanocrystals. Angew. Chem. Int. Ed..

[86] Zheng L, Qiu X, Zhang Z, Zhu D, Xu Y (2011). Solvothermal synthesis, crystal structure and luminescence property of a new 1D organic amine templated europium sulfate with helical chains. Inorg. Chem. Commun..

[87] Liu X, Gao H, Ward JE, Liu X, Yin B (2020). Power generation from ambient humidity using protein nanowires. Nature.

[88] Sun Y, Sun B, He J, Yang G, Wang C (2020). Millimeters long super flexible Mn_5_Si_3_@SiO_2_ electrical nanocables applicable in harsh environments. Nat. Commun..

[89] Aziz A, Zhang T, Lin Y-H, Daneshvar F, Sue H-J, Welland ME (2017). 1D copper nanowires for flexible printable electronics and high ampacity wires. Nanoscale.

[90] Sun H, Zhang Y, Zhang J, Sun X, Peng H (2017). Energy harvesting and storage in 1D devices. Nat. Rev. Mater..

[91] Lou Z, Shen G (2016). Flexible photodetectors based on 1D inorganic nanostructures. Adv. Sci..

[92] Wu W, Wang ZL (2016). Piezotronics and piezo-phototronics for adaptive electronics and optoelectronics. Nat. Rev. Mater..

[93] Song P, Qin H, Gao H-L, Cong H-P, Yu S-H (2018). Self-healing and superstretchable conductors from hierarchical nanowire assemblies. Nat. Commun..

[94] Qian H-S, Yu S-H, Gong J-Y, Luo L-B, Fei L-F (2006). High-quality luminescent tellurium nanowires of several nanometers in diameter and high aspect ratio synthesized by a poly(vinyl pyrrolidone)-assisted hydrothermal process. Langmuir.

[95] Liu J-W, Xu J, Hu W, Yang J-L, Yu S-H (2016). Systematic synthesis of tellurium nanostructures and their optical properties: from nanoparticles to nanorods, nanowires, and nanotubes. ChemNanoMat.

[96] Min-Seok K, Xing-Hua M, Ki-Hyun C, Seung-Yeol J, Kahyun H, Yun-Mo S (2018). A generalized crystallographic description of all tellurium nanostructures. Adv. Mater..

[97] Pine AS, Dresselhaus G (1971). Raman spectra and lattice dynamics of tellurium. Phys. Rev. B.

[98] Tao A, Kim F, Hess C, Goldberger J, He R, Sun Y, Xia Y, Yang P (2003). Langmuir–Blodgett silver nanowire monolayers for molecular sensing using surface-enhanced raman spectroscopy. Nano Lett..

[99] Tao AR, Huang J, Yang P (2008). Langmuir–Blodgettry of nanocrystals and nanowires. Acc. Chem. Res..

[100] Liu J-W, Wang J-L, Wang Z-H, Huang W-R, Yu S-H (2014). Manipulating nanowire assembly for flexible transparent electrodes. Angew. Chem. Int. Ed..

[101] Ariga K, Yamauchi Y, Mori T, Hill JP (2013). 25th anniversary article: what can be done with the langmuir-blodgett method? Recent developments and its critical role in materials science. Adv. Mater..

[102] Tian C, Ji H-P, Zong C-Y, Lu C-H (2015). Controlled fabrication of hierarchically microstructured surfaces via surface wrinkling combined with template replication. Chin. Chem. Lett..

[103] Baca AJ, Ahn J-H, Sun Y, Meitl MA, Menard E (2008). Semiconductor wires and ribbons for high-performance flexible electronics. Angew. Chem. Int. Ed..

[104] Xu F, Lu W, Zhu Y (2011). Controlled 3D buckling of silicon nanowires for stretchable electronics. ACS Nano.

[105] Fu X-W, Liao Z-M, Liu R, Xu J, Yu D (2013). Size-dependent correlations between strain and phonon frequency in individual ZnO nanowires. ACS Nano.

[106] Kleinman L (1962). Deformation potentials in silicon. I. Uniaxial strain. Phys. Rev..

[107] Du Y, Qiu G, Wang Y, Si M, Xu X, Wu W, Ye PD (2017). One-dimensional van der Waals material tellurium: Raman spectroscopy under strain and magneto-transport. Nano Lett..

[108] W. Harrison, *Elementary electronic structure* (*revised edition*). (World Scientific Publishing Company, 2004)

[109] Du Y, Maassen J, Wu W, Luo Z, Xu X, Ye PD (2016). Auxetic black phosphorus: a 2D material with negative Poisson’s ratio. Nano Lett..

[110] Jiang J-W, Chang T, Guo X, Park HS (2016). Intrinsic negative Poisson’s ratio for single-layer graphene. Nano Lett..

[111] Whang D, Jin S, Wu Y, Lieber CM (2003). Large-scale hierarchical organization of nanowire arrays for integrated nanosystems. Nano Lett..

[112] Kresse G, Furthmüller J (1996). Large-scale hierarchical organization of nanowire arrays for integrated nanosystems. Phys. Rev. B.

[113] Kresse G, Furthmüller J (1996). Efficient iterative schemes for ab initio total-energy calculations using a plane-wave basis set. Comput. Mater. Sci..

[114] Kresse G, Joubert D (1999). From ultrasoft pseudopotentials to the projector augmented-wave method. Phys. Rev. B.

[115] Klimeš J, Bowler DR, Michaelides A (2009). Chemical accuracy for the van der Waals density functional. J. Phys. Condens. Matter.

